# Exploring synthetic datasets for computer-aided detection: a case study using phantom scan data for enhanced lung nodule false positive reduction

**DOI:** 10.1117/1.JMI.11.4.044507

**Published:** 2024-08-07

**Authors:** Mohammad Mehdi Farhangi, Michael Maynord, Cornelia Fermüller, Yiannis Aloimonos, Berkman Sahiner, Nicholas Petrick

**Affiliations:** a FDA, CDRH, OSEL, Division of Imaging, Diagnostics, and Software Reliability, Silver Spring, Maryland, United States; b University of Maryland, Iribe Center for Computer Science and Engineering, Computer Science Department, College Park, Maryland, United States

**Keywords:** physical phantom, lung nodule detection, image transformation, CT scan, semi-supervised learning

## Abstract

**Purpose:**

Synthetic datasets hold the potential to offer cost-effective alternatives to clinical data, ensuring privacy protections and potentially addressing biases in clinical data. We present a method leveraging such datasets to train a machine learning algorithm applied as part of a computer-aided detection (CADe) system.

**Approach:**

Our proposed approach utilizes clinically acquired computed tomography (CT) scans of a physical anthropomorphic phantom into which manufactured lesions were inserted to train a machine learning algorithm. We treated the training database obtained from the anthropomorphic phantom as a simplified representation of clinical data and increased the variability in this dataset using a set of randomized and parameterized augmentations. Furthermore, to mitigate the inherent differences between phantom and clinical datasets, we investigated adding unlabeled clinical data into the training pipeline.

**Results:**

We apply our proposed method to the false positive reduction stage of a lung nodule CADe system in CT scans, in which regions of interest containing potential lesions are classified as nodule or non-nodule regions. Experimental results demonstrate the effectiveness of the proposed method; the system trained on labeled data from physical phantom scans and unlabeled clinical data achieves a sensitivity of 90% at eight false positives per scan. Furthermore, the experimental results demonstrate the benefit of the physical phantom in which the performance in terms of competitive performance metric increased by 6% when a training set consisting of 50 clinical CT scans was enlarged by the scans obtained from the physical phantom.

**Conclusions:**

The scalability of synthetic datasets can lead to improved CADe performance, particularly in scenarios in which the size of the labeled clinical data is limited or subject to inherent bias. Our proposed approach demonstrates an effective utilization of synthetic datasets for training machine learning algorithms.

## 1 Introduction

The recent development of intelligent systems and deep neural networks makes large-scale databases attractive for a variety of applications, including the diagnosis and detection of diseases in medical imaging. In medical applications, the dataset size can be limited, collecting data can be expensive, and the data may be subject to inherent bias slowing the progress of this new technology. The relatively small size of available labeled training datasets in medical imaging is a major bottleneck for methods based on supervised deep learning. This is mainly due to the higher cost of medical imaging annotations, which requires significant time and dedication by expert clinicians to establish reliable reference standards. As a result, methods that can increase the size of training datasets, such as synthetic datasets^1^ and weakly labeled datasets,^2^ are investigated to mitigate this limitation.

Synthetic datasets have the potential to offer numerous advantages in the research and development of machine learning algorithms applied in the healthcare domain.^3^ First, generating synthetic data could be more cost effective compared with approaches that attempt to cover clinical data population, both in terms of data collection and data annotation, especially for rare diseases or hard-to-reach populations. Second, demographic and health-related content in clinical datasets make it easier to identify individual subjects from real data, which can pose a risk to their privacy. Synthetic data, on the other hand, do not correspond to any individual subject and can be used without compromising privacy. Finally, although it can be challenging to obtain unbiased training data when there are inherent biases in the data collection process–stemming from under/over representation of different subgroups in clinical facilities–synthetic data may serve as an alternative or supplement to either filling gaps in data collection or enlarging samples for a subgroup.

Synthetic imaging data can be obtained using different approaches; knowledge-based approaches involve the use of mathematical equations, analytical models, and computer simulations to generate data that mimic real imaging modalities. They rely on principles of physics, imaging protocols, and simplifying assumptions.^4^^,^^5^ Although these techniques focus on modeling imaging systems, other forms of synthetic data, such as physical phantoms, explore data generation methods that target modeling patients and their associated conditions. These phantoms typically consist of tangible objects or materials with known properties that are designed to simulate patients with specific medical conditions, providing controlled and calibrated synthetic data, making them potentially valuable for quality assurance, calibration, and algorithm training and evaluation purposes.^6^ Deep learning-based approaches are another means that employ deep neural networks, such as generative adversarial networks (GANs)^7^ and autoencoders,^8^ to learn from datasets of images and generate synthetic data that closely resembles the real data.^9^

Training robust machine learning models for medical imaging applications often faces the challenge of only having a limited set of real-world data. In this paper, we study the potential of using scans of physical phantoms acquired using clinical computed tomography (CT) imaging systems to obtain labeled data for training neural network classifiers. Our study aims to address the performance gap of machine learning models that are trained on limited-size clinical training datasets. By incorporating scans of synthetic abnormalities within the physical phantom as part of the training data, we enhance the algorithm’s ability to learn the characteristics of target abnormalities. Simultaneously, including an unlabeled dataset of clinical scans can be used to complement the physical phantom data to expose the neural network to clinical data and increase the variability in the training set. By combining the two sources of data, our proposed algorithm can be trained at a lower cost while exposing it to cases expected to be found in the clinical settings.

## 2 Materials and Methods

**Fig. 1:**
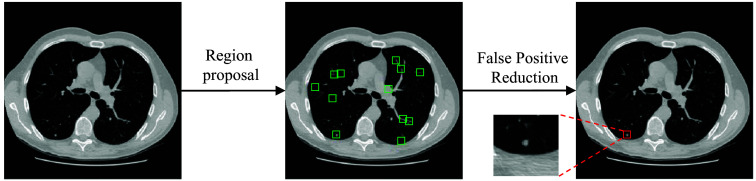
Overview of our lung nodule detection framework. The initial stage involves region proposal, which parses the volumetric CT scan with high sensitivity to identify potential nodule locations. Subsequently, the second stage aims for high precision, focusing on eliminating FPs generated in the first stage by classifying the ROIs defined by the initial stage detections as nodule or non-nodule regions.

We investigate the effectiveness of including image data from scans of synthetic physical phantoms in training neural networks within the false positive (FP) reduction stage of a multi-stage computer-aided detection (CADe) system. In this system, shown in Fig. 1, an initial stage of the region proposal parses the volumetric scans to identify suspicious nodule locations. The initial stage prioritizes high sensitivity to capture diverse nodule types, often leading to many FPs. Subsequently, an FP reduction network operates in the second stage by classifying regions of interest (ROIs) defined by the initial stage detections as nodule or non-nodule regions. This second stage refines the identified candidate locations with the goal of more precisely separating true nodules from FPs.

### 2.1 Datasets

#### 2.1.1 Phantom and synthetic nodules

**Fig. 2:**
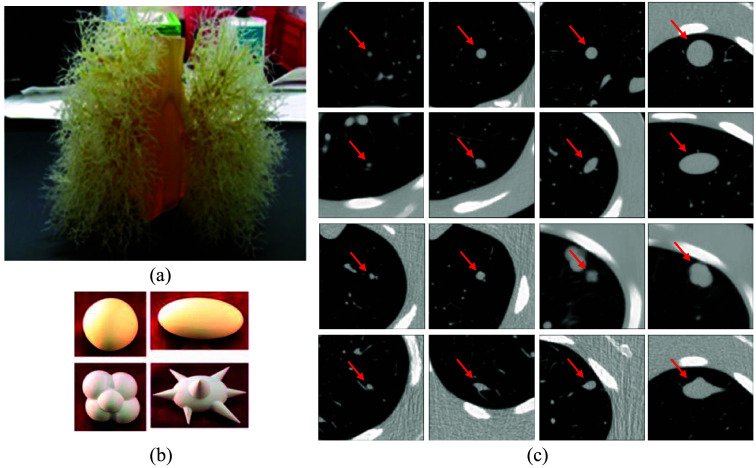
(a) Photograph of the thoracic phantom with the vasculature insert attached. (b) Examples of different synthetic nodule shapes. Clockwise from upper left: spherical, elliptical, spiculated, and lobulated nodules.^10^ (c) CT scan slices of synthetic nodules with a variety of shapes and sizes. From top to bottom, each row represents nodules in spherical, elliptical, lobulated, and spiculated shapes. Columns from left to right represent nodules of diameters 5, 8, 10, and 20 mm. All images were shown for the in-plane ROI size of 75 mm and the window level of −1000 to 400 HUs.

The synthetic database used in this study was obtained by imaging a physical anthropomorphic thoracic phantom^6^ with a vascular insert attached as shown in Fig. 2(a). In the construction of this phantom, synthetic pulmonary nodules [Fig. 2(b)] were placed in pre-defined positions either attached to synthetic vessels or suspended in foam in a non-attached configuration. These nodules vary in size, shape, and density to model a variety of potential nodules in the clinical setting. The phantom with the inserted nodules was scanned using a 16-detector row helical CT system (Philips, Mx8000 IDT) with varying combinations of nodule layouts, doses, and pitches; slice collimation; and then reconstruction using various combinations of slice thicknesses and reconstruction kernels.^10^ In total, 48 synthetic nodules including nodules with sizes ranging between 5 and 20 mm diameter; different shapes including spherical, elliptical, lobulated, spiculated; and densities of −630, −10, 100 HUs were used in this study. Figure 2(c) shows photographs of 16 nodules of varying sizes and shapes with a density equal to −10 HUs. The remaining 32 nodules consist of identical shapes and sizes but with lower (−630  HU) and higher (100 HU) densities.

**Fig. 3:**
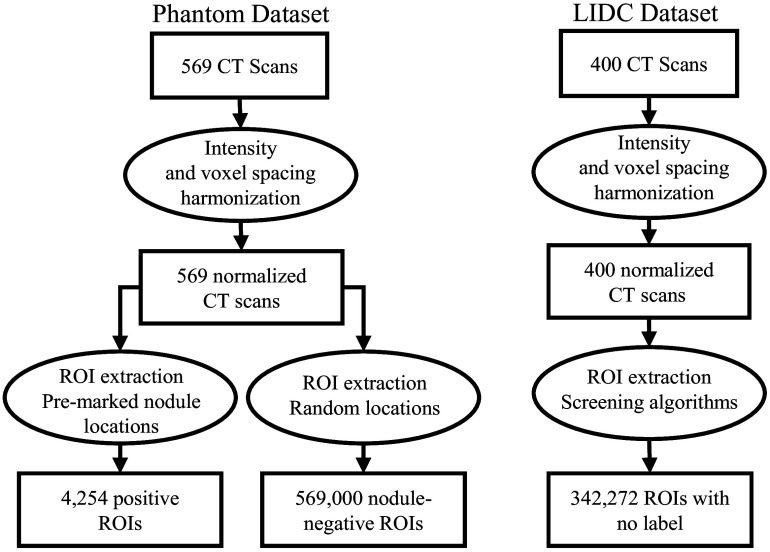
Training data pre-processing and ROI extraction.

Figure 3 shows the process of obtaining training data from this database. To ensure consistent pixel spacing across the scans in these datasets, a normalization step, in which all scans are normalized to have a uniform 0.625  mm×0.625  mm in-plane pixel size and a slice spacing of 2 mm, is performed. In addition, the HU intensities are clipped to the range of [−1000,400] and rescaled to [−1.0,1.0]. From a total of 569 physical phantom nodule layouts and scans,^10^ 4254 ROIs of size $30\;{\mathrm{mm}}^{3}$ are extracted and form the nodule-positive synthetic training samples. Negative ROIs (ROIs without nodules) are obtained by randomly sampling the remaining areas of each phantom scan, such that they do not overlap with any inserted synthetic nodule location. Each scan is sampled 1000 times in this manner to obtain a total of 569,000 negative candidates across the phantom scan dataset. To account for the imbalance among positive and negative ROIs, the nodule-positive ROIs are over-sampled 250 times.^11^

#### 2.1.2 LIDC-IDRI database

In addition to images obtained from scanning the physical phantom, the proposed method incorporates clinical data into the training in a semi-supervised fashion in which unlabeled clinical data and labeled phantom data were included in the algorithm training. This is motivated by the intention to mitigate the labor-intensive effort associated with clinical data annotations while still exposing the neural network to a diverse set of abnormalities. To achieve this, unlabeled CT scans from the Lung Image Database Consortium and Image Database Resource Initiative (LIDC-IDRI)^12^ were included as part of the training data. The LIDC-IDRI dataset is a publicly available database consisting of 1018 cases of clinical thoracic CT scans. Although the LIDC-IDRI dataset contains lesion labels, we utilized this data in an unlabeled manner as part of our training process to better assess the potential benefits of including phantom scan data and limiting the need for clinical data labeling. We processed this database following the same filtering criteria as the LUNA16 challenge.^13^ This preprocessing involved filtering out scans with slice thicknesses greater than 2.5 mm, inconsistent slice spacing, or missing slices. After filtering, 888 CT scans remained; of these, 400 were selected as unlabeled clinical training data. To ensure consistent pixel spacing and intensity values across the datasets, the LIDC-IDRI scans are normalized in a similar fashion to the phantom database and as shown in Fig. 3. To obtain the ROIs from this dataset, we used the candidates provided by the LUNA16 challenge. These candidates are the results of screening the scans by five different detectors^13^ and combining their outputs. These algorithms were developed using hand-engineered features including shape index and curvedness local features;^14^ intensity, shape, and texture features;^15^ thresholding and morphological operations;^16^ nodule enhancement engineered filters;^17^ and region growing with adaptive threshold.^18^ The combination of these carefully designed hand-crafted features, each designed to target specific types of nodules, such as juxta-pleural, juxta-vascular, solid, and non-solid nodules, resulted in ∼750 candidate ROIs per scan. We extracted $30\;{\mathrm{mm}}^{3}$ ROIs centered at each candidate location, again to match the process used with the phantom data.

The remaining 488 scans in this database are split into two sets for tuning (88 scans) and the final evaluation dataset (400 scans); each scan in this database was annotated by four radiologists, in a two-stage process: in the first stage, each of the four annotators annotates the scan independently; in the second stage, annotators view the annotations for all annotators and reassess. This process produces higher quality annotations and allows for a robust benchmarking of the proposed algorithm. Following the nodule selection in the LUNA16 challenge, the nodules in the testing set are filtered according to diameter >3  mm and annotated by >3 of four annotators. Findings that do not pass this filtering are excluded from consideration, and their detection is not counted as either FP or true positive.

Table 1 summarizes the distribution of training and testing scans, derived from the LIDC-IDRI and phantom databases. The CT scans in the phantom database are used solely for training, whereas the LIDC-IDRI scans are divided into three non-overlapping sets: unlabeled training, labeled tuning, and labeled test sets. As mentioned above, annotations were available for the entire LIDC-IDRI database, but the 400 scans assigned to the “training unlabeled” set were used without labels to investigate the impact of unlabeled clinical datasets in mitigating distributional differences between the training and testing data.

**Table 1:** Number of CT scans from the LIDC-IDRI and anthropomorphic phantom datasets. Phantom scans are exclusively used for training.

	Training labeled	Training unlabeled	Tuning	Test	Total
LIDC-IDRI	—	400	88	400	888
Phantom	569	—	—	—	569

The LIDC-IDRI dataset represents clinical data, with non-overlapping scans allocated for unlabeled training, labeled tuning, and labeled test for the evaluation of the method.

### 2.2 Training Framework

We build our FP reduction network via a semi-supervised learning framework and a combination of stochastic augmentations. The unlabeled clinical data in our training set aim to help the network learn the characteristics of a variety of abnormalities actually observed in the clinical data that go beyond the simple shapes found in our phantom. The augmentations, on the other hand, are introduced to enrich and add variability into the training set and better account for the visual appearance of the abnormalities in the phantoms as they are overly simplified compared with clinical data.

**Fig. 4:**
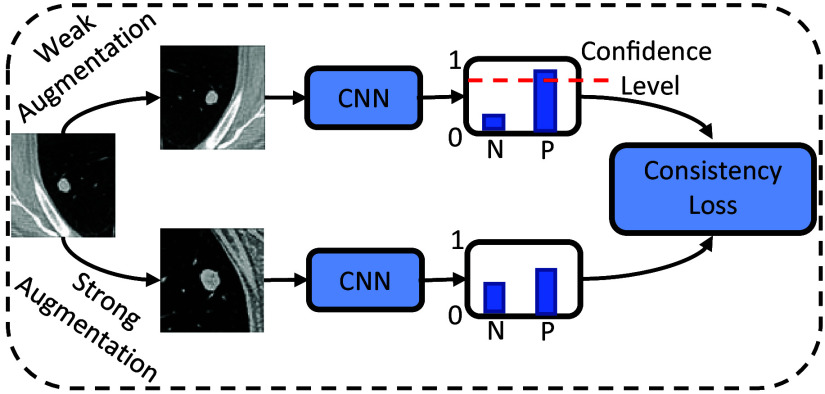
Diagram of the consistency loss ($l_{u}$) in Eq. (1). The unlabeled stream of data is passed through different strengths of augmentation, and the network is updated for consistent predictions.

The training framework is inspired by the consistency prediction introduced in the fixMatch.^19^ Specifically, the proposed loss function to update the neural network weights is defined on two streams of labeled and unlabeled datasets:

$$L=l_{s}+\lambda_{1}l_{u}+\lambda_{2}l_{p},$$ (1)

where $l_{s}$ corresponds to the loss term defined on the labeled stream of data and functioning as a conventional fully supervised loss function. The second term in Eq. (1) is the consistency loss ($l_{u}$) defined on the unlabeled dataset, which ensures that predictions for unlabeled data remain consistent across different transformations on input ROIs. This is achieved by passing the unlabeled stream of data through a set of weak augmentation operations and a set of strong augmentation operations, as shown in Fig. 4. The weak and strong augmentations used in this work are defined below. Predictions obtained from each transformation are forced to agree by the consistency loss in this figure. To ensure that the network updates based on the consistency loss occur for samples in which the network predictions are stable, this term is applied only to ROIs with predictions in the weakly augmented path that surpass a confidence score empirically set to 0.95. This allows the neural network to rely primarily on labeled data in cases in which predictions on unlabeled data have a lower confidence. For example, in the initial stages of training when the network predictions are closer to random guessing, the network is primarily driven by the labeled data. However, as training progresses and the network predictions on unlabeled data achieve higher levels of confidence, the contribution from the consistency loss is activated.^19^

In addition to the previous loss terms, the samples in the unlabeled stream are assigned pseudo-labels prior to the training, as shown in the third term of Eq. (1) ($l_{p}$); the neural network^11^ fully trained on physical phantom data is applied to the unlabeled dataset, and the samples with a prediction score above a threshold are labeled as nodule and non-nodule, allowing the model to learn directly from the unlabeled data and improve its predictions. To avoid noise in the assigned pseudo-labels, a high threshold value is determined empirically with the values of 0.01 and 0.99 assigning the negative and positive pseudo-labels, respectively.

**Fig. 5:**
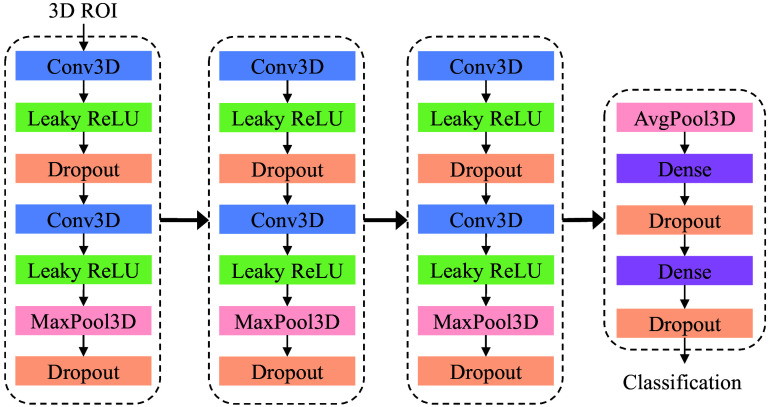
Architecture of the FP reduction classification network.^11^ Three blocks of convolutional layers are applied consecutively followed by two fully connected layers.

The neural network architecture for the FP reduction application task is identical to the classification network presented in our previous work.^11^ The architecture of this network is presented in Fig. 5 and consists of three convolutional and one fully connected blocks. Each convolutional block consists of two 3D convolutions, each followed by a Leaky ReLU activation function. The outputs from the second convolution layers in each block are sub-sampled through 3D Max Pooling. The dense block consists of two fully connected layers, preceded by 3D average pooling. We selected the hyperparameters of this network identical to our previous work,^11^ which include 32, 64, and 128 kernels of size 3×3×3 in each convolutional block, respectively. Within each block, dropout layers with a rate of 0.3 are applied during training. In the dense block, each fully connected layer consists of 128 nodes, and during training, a dropout value of 0.9 is used. The only adjustment to the training hyperparameters is introduced in the loss term controlling the contribution of the labeled and unlabeled stream of data. We derive, from our tuning set, the parameters $\lambda_{1}=0.5$ and $\lambda_{2}=0.5$ to update the network parameters during the training phase. The higher value of $\lambda_{1}$ in proportion to $\lambda_{2}$ implies that the network relies more on the labeled dataset to update its weights during training. In addition, we introduce a weight decay over the kernel weights as a regularizer to improve the training stability.^11^ The classification loss terms of $l_{s}$, $l_{u}$, and $l_{p}$ in Eq. (1) are implemented using focal loss:^20^

$$\mathrm{FL}(p,y)=-\alpha (1-p_{t}{)}^{\gamma }\;\log(p_{t}).\;\text{where}\;p_{t}=\{\begin{array}{ll} p & \text{if}\;y=1\\ \left(1-p\right) & \text{otherwise} \end{array}.$$ (2)

In Eq. (2), p corresponds to network predictions, and y corresponds to the ground truth or pseudo-labels. This function focuses the training on less-represented examples by adjusting the parameter α, empirically set to 16.0. The parameter γ (empirically set to 2.0) controls the degree of emphasis on hard examples, i.e., samples in which the network assigns less confident scores. This function can be seen as a generalized form of the binary cross-entropy function, in which setting α and γ to 1 simplifies the function to a standard binary cross-entropy.

**Fig. 6:**
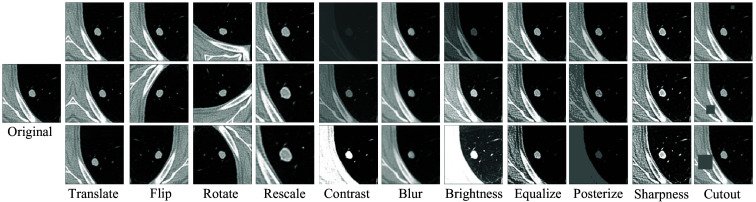
Visualization of the parameterized augmentations. Each column illustrates three instances of the augmentation. Each image shows the central slice of the transformed volumetric image. The “translate” and “flip” augmentations are referred to as weak augmentations in the unlabeled data stream. When followed by any of the other augmentations, they are referred to as strong augmentations.

The training framework employs a range of augmentations in both the labeled and unlabeled data streams, each parameterized and applied with varying strengths during training. Figure 6 shows these augmentations, displaying three versions of the perturbed image with different augmentation strengths. In the unlabeled data stream, the flip and translate augmentations, shown as weak augmentations in Fig. 4, serve as the baseline for the consistency loss computations. Following a weak augmentation, additional augmentations from Fig. 6 are randomly selected, transforming the image further; these are termed as strong augmentations in Fig. 4. In the labeled data stream, with the addition of “identity transformation” representing no operation, these augmentations are applied uniformly across all input samples—both positive and negative ROIs—selected at random with equal probability. This enriches the training dataset, exposing the model to diverse data variations and better utilization of the training data.

## 3 Experimental Results

We benchmark the performance of the proposed algorithm with two experiments. First, we study the performance of the algorithm when trained only on physical phantom and unlabeled clinical data and evaluate it on clinical scans obtained from the LIDC-IDRI database. The second experiment illustrates the potential benefit of using physical phantom data to enlarge a small clinical labeled dataset derived from the LIDC-IDRI database.

### 3.1 Training on Phantom and Unlabeled Clinical Scans

**Fig. 7:**
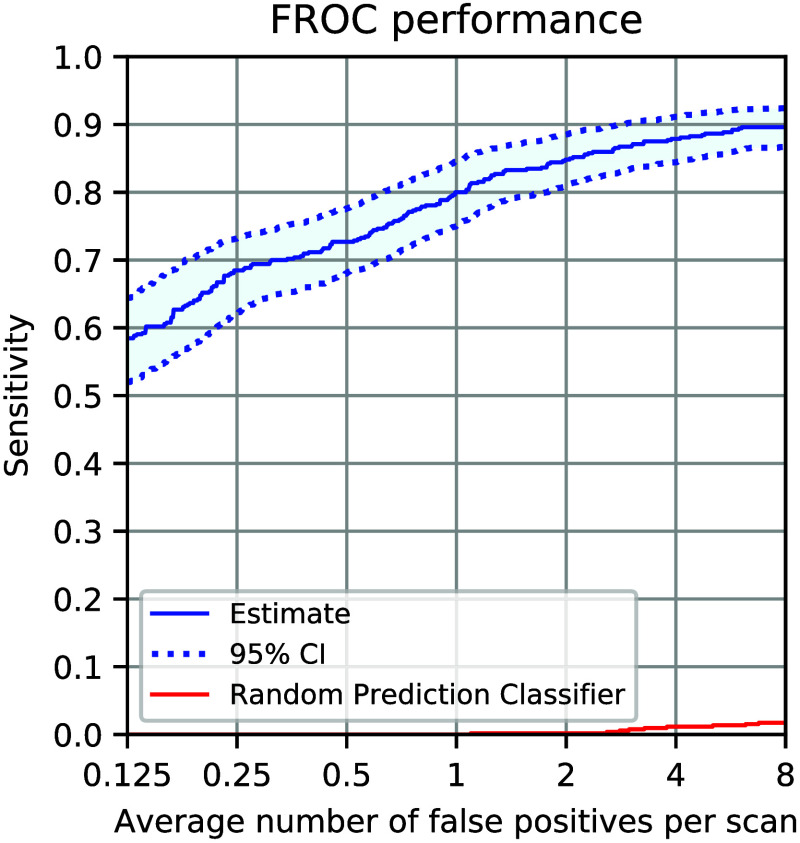
Free response operating characteristic (FROC) performance of the algorithm trained on synthetic data, unlabeled clinical data and augmentations. The performance is on test data from 400 clinical LIDC-IDRI scans. For reference, the performance of a random prediction classifier is shown with the red curve.

This section reports on the experimental results when the labeled training data are obtained from the physical phantom as outlined in Sec. 2.1.1. The algorithm is evaluated on a test set that consists of 400 clinically scanned subjects from the LIDC-IDRI database, containing a total of 520 nodules. For this testing dataset, an average of 750 candidates per scan was generated using the algorithms detailed in Sec. 2.1.2. In reporting the performance, we count a candidate location as a detection success (true positive) if the center of the detection lies within the boundary of annotations provided in the reference standard. The effectiveness of the proposed method is assessed using the free receiver operating characteristic (FROC) curve and is summarized by the competition performance metric (CPM).^21^ The CPM measures the average sensitivity at seven operating points of the FROC curve: $\frac{1}{8}$, $\frac{1}{4}$, $\frac{1}{2}$, 1, 2, 4, and 8 FPs per scan. Figure 7 shows the FROC performance of the proposed training method, including augmentations and unlabeled data [please refer to Eq. (1)], indicating a CPM value of 0.773. For reference, the figure also includes the performance of a random prediction classifier on this set of candidates, represented by the red curve. This classifier assigns each nodule candidate a random confidence score between 0 and 1. Given the heavily imbalanced nature of positive and negative candidates in the testing set, with about 750 FPs per scan, the random score assignment leads to a significantly lower performance. The comparison between the two curves highlights the discrimination power gained by training the neural network using labeled phantom scan data.

**Fig. 8:**
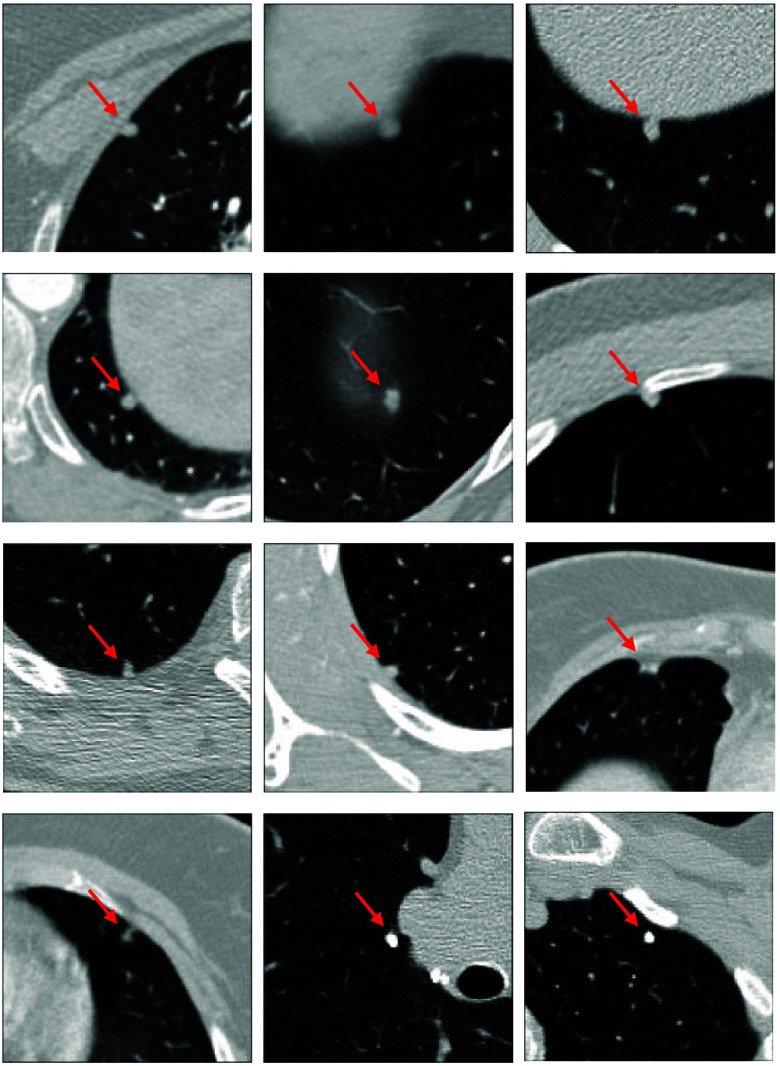
Central slices of nodules that were missed by the algorithm. In all images, the nodule is centered in an ROI of an in-plane dimension of $75\;{\mathrm{mm}}^{2}$. Images are shown with the same window level of −1000 to 400 HU.

The results in Fig. 7 show that the algorithm achieved a relatively high sensitivity of 80% at one and 90% at eight FPs per scan. Given the limited clinical relevance of a higher number of FPs, the evaluation in this figure focuses on the range of FP rates of up to eight FPs per scan. However, we noted that the algorithm failed to detect and retrieve 12 nodules from the list of available candidates even at 250 FPs per scan. Figure 8 shows the central slice images of all 12 false negatives. The failure to detect the two calcified nodules—shown in the bottom row of the figure—may be attributed to their high density as the synthetic nodules in our training phantom dataset^6^ were set to a maximum density of 100 HU as detailed in Sec. 2.1.1. The remaining false negatives all represent small nodules (<7  mm in diameter) that are either attached to the chest wall or obscured by other organs. The occurrence of these false negatives may be attributed to the under-representation of nodules with attachment to the pleural surface and near other organs as they were not part of the anatomical context specifically modeled in the phantom scans.^6^

**Fig. 9:**
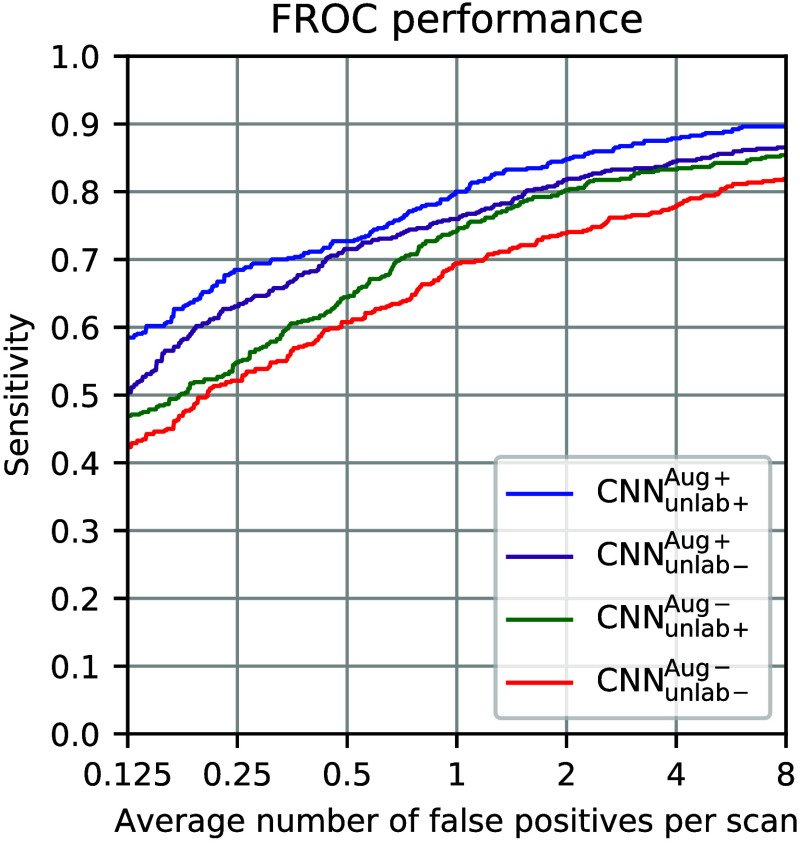
FROC performance for different scenarios of training using the phantom CT data: ${\mathrm{CNN}}_{\text{Unlab}-}^{\mathrm{Aug}-}$ shows the baseline FROC performance when training was performed by flip, rotation, and translation augmentations without the inclusion of unlabeled clinical scans, resulting in CPM performance = 0.654. ${\mathrm{CNN}}_{\text{Unlab}+}^{\mathrm{Aug}-}$ shows the performance when unlabeled clinical scans were added to the baseline training framework with CPM = 0.698. ${\mathrm{CNN}}_{\text{Unlab}-}^{\mathrm{Aug}+}$ shows the performance when training data were enriched with all of the proposed augmentations without inclusion of unlabeled data with CPM = 0.734. ${\mathrm{CNN}}_{\text{Unlab}+}^{\mathrm{Aug}+}$ shows the performance of the proposed approach with the summary performance of CPM = 0.773.

Figure 9 shows the impact of parameterized augmentations and the inclusion of the unlabeled dataset in the final model’s performance. For this experiment, the training data are passed through translation, rotation, and flip augmentations, which are the typical augmentation methods for the lung nodule detection applications, referred to as ${\mathrm{CNN}}^{\mathrm{Aug}-}$. In comparison, ${\mathrm{CNN}}^{\mathrm{Aug}+}$ shows the performance of the network when all transformations introduced in Sec. 2 are applied during training. In the second pair of comparisons, the neural network is trained when the unlabeled clinical scans are deleted from the training set (${\mathrm{CNN}}_{\text{Unlab}-}$), and it is compared against a training set in which the LIDC-IDRI scans are included as unlabeled data, ${\mathrm{CNN}}_{\text{Unlab}+}$. Note that ${\mathrm{CNN}}_{\text{Unlab}+}^{\mathrm{Aug}+}$ is the performance curve shown in Fig. 7.

The comparison between the ${\mathrm{CNN}}_{\text{Unlab}-}^{\mathrm{Aug}-}$ and ${\mathrm{CNN}}_{\text{Unlab}-}^{\mathrm{Aug}+}$ performances in Fig. 9 shows that training with the parameterized augmentations resulted in a higher performance with the performance summary of CPM = 0.654 versus CPM = 0.734. We estimated the confidence interval (CI) of the CPM difference of the two training strategies by bootstrapping the difference in CPM 1000 times. The estimated 95% CIs of the CPM difference of the training strategies was [0.052, 0.102], confirming a statistically significant improvement for ${\mathrm{CNN}}_{\text{Unlab}-}^{\mathrm{Aug}+}$ compared with ${\mathrm{CNN}}_{\text{Unlab}-}^{\mathrm{Aug}-}$. Adding the unlabeled CT scans of the LIDC-IDRI database to the training ${\mathrm{CNN}}_{\text{Unlab}+}^{\mathrm{Aug}+}$ also led to a higher detection performance as shown in the figure, with a CPM score of 0.773 compared with a CPM score of 0.734 for ${\mathrm{CNN}}_{\text{Unlab}-}^{\mathrm{Aug}+}$. We estimated the 95% CI of the CPM difference of the two training strategies (${\mathrm{CNN}}_{\text{Unlab}-}^{\mathrm{Aug}+}$ versus ${\mathrm{CNN}}_{\text{Unlab}+}^{\mathrm{Aug}+}$) as [0.024, 0.055], thereby confirming that the improvement obtained with the inclusion of unlabeled LIDC-IDRI data was statistically significant. These improvements are attributed to the simplified nature of the phantom data that is enhanced by the stochastic augmentations and the inclusion of the clinical data within the training framework.

### 3.2 Enlarging a Labeled Training Set with Phantom Scans

To illustrate the effectiveness of including phantom scans to expand a small clinical dataset, we conducted a set of experiments that combined a number of labeled baseline clinical scans with phantom scans during the training process. In these experiments, the baseline labeled training sets consist of an increasing number of labeled LIDC-IDRI scans, and the algorithm test performance is reported based on the same 400 scans as outlined in the previous section. In the first set of experiments, we train the classification network using augmentations of flip, rotation, and translation (i.e., ${\mathrm{CNN}}_{\text{Unlab}-}^{\mathrm{Aug}-}$). The results of these experiments are reported in Table 2. Each row of the table reports the sensitivity and CPM performance when the given number of LIDC-IDRI scans is used for training, followed by the results when the training datasets are expanded with 569 phantom scans. In consecutive rows, we incrementally add more scans from the LIDC-IDRI database into the training.

**Table 2:** Performance of the FP reduction algorithm trained with flip, translation, and rotation augmentations, ${\mathrm{CNN}}_{\text{unlab}-}^{\mathrm{Aug}-}$.

Training scans		Sensitivity at different FPs per scan							CPM	95% CI
LIDC-IDRI	Phantom	0.125	0.25	0.5	1	2	4	8		
5	—	0.468	0.557	0.623	0.662	0.717	0.756	0.789	0.653	(0.037, 0.125)
	569	**0.517**	**0.606**	**0.688**	**0.759**	**0.815**	**0.859**	**0.894**	**0.734**	
25	—	0.553	0.625	0.674	0.721	0.751	0.793	0.830	0.707	(0.036, 0.113)
	569	**0.569**	**0.665**	**0.746**	**0.813**	**0.863**	**0.891**	**0.910**	**0.780**	
50	—	0.578	0.668	0.726	0.778	0.814	0.853	0.875	0.756	(0.032, 0.088)
	569	**0.624**	**0.700**	**0.789**	**0.847**	**0.892**	**0.912**	**0.936**	**0.814**	
100	—	**0.695**	**0.767**	**0.813**	0.864	0.890	0.903	0.920	**0.836**	(−0.031, 0.012)
	569	0.641	0.722	0.790	**0.866**	**0.898**	**0.929**	**0.946**	0.827	
400	—	**0.775**	**0.848**	**0.898**	**0.926**	**0.947**	**0.968**	**0.973**	**0.905**	(−0.029, −0.008)
	569	0.723	0.813	0.878	0.921	0.939	0.962	0.970	0.886	

Each row represents the test performance when different numbers of LIDC-IDRI scans are selected for training and compared against a training set that is enlarged with 569 phantom scans. The comparison is reported in terms of sensitivities at different FPs per scan and the overall CPM. The scores of the best model in each comparison pair are shown in bold. The last column shows the 95% CI of the CPM difference of the two training strategies (i.e., with and without phantom scans).

The results demonstrate that augmenting small-sized LIDC-IDRI training data (i.e., 5, 25, and 50 scans) with phantom scan data improves all of the performance metrics. However, this improvement diminishes as more clinical LIDC-IDRI scans are added to the labeled training data. Furthermore, the experiments reveal that including training phantom scans produces a greater sensitivity improvement at higher numbers of FPs per scan, compared with the relatively smaller improvement observed at lower FP rates in most of our experiments. For example, when only five LIDC-IDRI scans are used for training, the improvement in sensitivity at 8 FPs/scan is 10.5% (0.894 to 0.789), whereas the improvement is only 4.9% (0.517 to 0.468) at 0.125 FPs/scan. This trend, observed to some extent in all experiments, ultimately resulted in a lower overall CPM performance with the addition of phantom scans in the training set (i.e., 400 LIDC-IDRI scans in the last row of the table). This trend is likely a result of the simplified appearance of phantom scans, particularly concerning negative/FPs ROIs. Unlike the negative ROIs extracted from the LIDC-IDRI database, which represents various anatomical structures and hard mimics such as blood vessels, airways, and scar tissues, the negative ROIs from the phantom scans lack such diversity in anatomy. These hard mimics may exhibit appearances similar to lung nodules in CT images, making their discrimination from true positives challenging for the neural network classifier. Consequently, during training, the classifier may become overwhelmed by the substantial number of simple phantom FPs when an abundance of these samples serves as negatives in the training data. This results in a network with less discriminatory power between true positives and more challenging FPs compared with a network trained on a larger number of labeled clinical scans.

## 4 Discussion

In this study, we introduced a novel method for training neural networks using CT scans obtained from a physical anthropomorphic phantom. To address the challenge posed by the simplified appearances of the manufactured lung nodules in the phantom, we conducted an investigation into the potential benefits of enriching the training dataset. This enrichment involved the incorporation of a broad range of randomized and parameterized augmentations. In addition, we explored the integration of unlabeled clinical data, providing an alternative when data annotations are prohibitively expensive or challenging to obtain.

Our experimental results demonstrate the promise of training neural networks with, or partially with, physical phantom scan data; using our proposed method, a CPM performance of 0.820 was obtained with only 25 labeled clinical scans (the second row in Table 3). This performance surpassed the baseline framework even when the size of the training set in the baseline was increased to 50 labeled clinical CT scans (the third row in Table 2, CPM = 0.756) and was only slightly worse than the performance when the size of the labeled training set increased to 100 scans (the fourth row of Table 2, CPM = 0.836).

**Table 3:** Performance of the FP reduction algorithm trained with all augmentations and when including the unlabeled clinical data, ${\mathrm{CNN}}_{\text{unlab}+}^{\mathrm{Aug}+}$.

Training scans		Sensitivity at different FPs per scan							CPM	95% CI
LIDC-IDRI	Phantom	0.125	0.25	0.5	1	2	4	8		
5	—	**0.592**	**0.662**	**0.772**	0.777	0.822	0.861	0.888	0.761	(−0.014, 0.040)
	569	0.574	0.658	0.742	**0.800**	**0.849**	**0.884**	**0.903**	**0.773**	
25	—	0.640	0.716	**0.786**	0.830	0.864	0.899	0.910	0.806	(−0.011, 0.039)
	569	**0.661**	**0.717**	0.777	**0.850**	**0.897**	**0.913**	**0.922**	**0.820**	
50	—	**0.703**	**0.773**	**0.830**	0.870	0.908	0.920	0.940	**0.849**	(−0.035, 0.009)
	569	0.661	0.733	0.791	**0.871**	**0.915**	**0.939**	**0.949**	0.837	
100	—	**0.723**	**0.780**	**0.858**	**0.910**	0.923	0.936	0.954	**0.869**	(−0.034, 0.002)
	569	0.674	0.747	0.823	0.883	**0.924**	**0.943**	**0.961**	0.851	
400	—	**0.757**	**0.844**	**0.897**	**0.933**	**0.955**	0.963	**0.971**	**0.903**	(−0.027, 0.003)
	569	0.730	0.814	0.871	0.920	0.951	**0.965**	0.971	0.889	

Each row represents the performance when different numbers of LIDC-IDRI scans are selected for training and compared against a training set that is enlarged with 569 phantom scans. The comparison is reported in terms of sensitivities at different FPs per scan and the overall CPM. The scores of the best model in each comparison pair are shown in bold. The last column shows the 95% CI of the CPM difference of the two training strategies (i.e., with and without phantom scans).

The contribution of the phantom data was more pronounced when training was performed with traditional augmentations, such as flip, rotation, and translation (${\mathrm{CNN}}^{\mathrm{Aug}-}$), as shown in Table 2. This was primarily evident in the reported 95% CIs calculated for the CPM difference between the two training strategies. For example, the improvement obtained by adding phantom scans to the training set of 50 clinical scans had a CI of (0.032, 0.088). However, as the number of clinical scans increased or training was conducted with a stronger set of augmentations, the benefit of the phantom dataset diminished, as indicated by the paired comparisons shown in Table 3. This trend could be attributed to the variability present in the training set; when there is limited variability due to a smaller number of training samples or restricted augmentation, the added benefit of phantom scans is more pronounced. Although some experiments in Table 3 show slight improvements in the CPM performance, these improvements remain too small to draw any substantive conclusions.

To evaluate the performance of our neural network compared with existing methods, we conducted an additional study using the LIDC-IDRI database. Here, the entire clinical database, consisting of 888 scans, was divided into 10 folds, and 10-fold cross-validation was employed to assess the network’s performance across the entire dataset. This setup aligns with established practices in the literature^22^^–^^24^ for comparing our neural network with existing methods. The 10-fold cross-validation yielded a CPM score of 0.915, a performance level comparable to state-of-the-art results in this application and clinical data.^22^^–^^24^

## 5 Conclusion

Synthetic data, such as physical anthropomorphic thorax phantoms, may help reduce the number of labeled clinical datasets needed for training neural networks. In this work, we investigated a novel method to expand clinical datasets using data containing synthetic lung nodules to effectively expand the training set and improve the performance for CADe systems. To address distribution differences between clinical and phantom data, in which phantom and synthetic nodules represent simplified versions of clinical lung CT data, our method incorporates image augmentations. These augmentations introduce perturbations and enhance variability in the appearances of abnormalities. Furthermore, our approach adopts a semi-supervised learning approach, exposing the neural network to real clinical data without associated labels. This integration brings the advantages of real-world data variability and characteristics to the training process without incurring substantial annotation costs. The combination of these three strategies shows real promise in overcoming challenges related to a small training dataset and the costly annotation of medical data.

## Data Availability

The data that support the findings of this study are available from the corresponding author upon reasonable request.
